# Comparison of Del Nido Cardioplegia and Blood Cardioplegia in Terms of Development of Postoperative Atrial Fibrillation in Patients Undergoing Isolated Coronary Artery Bypass Grafting

**DOI:** 10.21470/1678-9741-2020-0047

**Published:** 2021

**Authors:** Umut Serhat Sanrı, Kadir Kaan Özsin, Faruk Toktaş, Şenol Yavuz

**Affiliations:** 1 Department of Cardiovascular Surgery, University of Health Sciences, Bursa Yuksek Ihtisas Training and Research Hospital, Bursa, Turkey.

**Keywords:** Cardioplegic Solutions, Coronary Artery Bypass., Cardiac Surgical Procedures, Atrial Fibrillation, Troponin T, Heart Arrest, Induced

## Abstract

**Objective:**

Del Nido cardioplegia (DNC) has been used in pediatric cardiac surgery for many years with a single dose application and its usage in adult cardiac surgery has been increasing in recent years, with results being published. In this study, we aimed to investigate the effect of DNC on the development of postoperative atrial fibrillation (PoAF).

**Methods:**

In this retrospective observational comparative study, 255 patients who underwent isolated on-pump coronary artery bypass grafting, between January 2019 and November 2019, were enrolled. The patients were divided into two groups: DNC (n=132) and blood cardioplegia (BC) (n=123). Intraoperative and postoperative data were evaluated and compared in terms of the development of PoAF.

**Results:**

We found that the development of PoAF and the length of hospital stay remain significantly higher in the BC group (P=0.044, P<0.001, respectively). In addition, the aortic cross-clamp time and the cardioplegia volume delivered were significantly lower in the DNC group (*P*=0.042, *P*<0.001, respectively). In multivariate logistic regression analysis, only higher cardioplegia volume was determined as an independent predictor for PoAF development (OR 1.001; 95% CI 1.000-1.001; *P*=0.033). We did not found difference between groups in terms of troponin T, inotropic drug support, need for intraaortic balloon pump and mortality.

**Conclusion:**

This study showed that DNC can be used safely in adult coronary bypass surgery and PoAF development effect is reduced.

**Table:** 

Abbreviations, acronyms & symbols				
**AF**	**= Atrial fibrillation**		**HT**	**= Hypertension**
**BC**	**= Blood cardioplegia**		**IABP**	**= Intraaortic balloon pump**
**CABG**	**= Coronary artery bypass grafting**		**ICU**	**= Intensive care unit**
**CI**	**= Confidence interval**		**OR**	**= Odds ratio**
**COPD**	**= Chronic obstructive pulmonary disease**		**PoAF**	**= Postoperative atrial fibrillation**
**CRP**	**= C-reactive protein**		**ROS**	**= Reactive oxygen species**
**CPB**	**= Cardiopulmonary bypass**		**SPSS**	**= Statistical Package for the Social Sciences**
**DNC**	**= Del Nido cardioplegia**		**STS**	**= Society of Thoracic Surgeons**
**ECG**	**= Electrocardiography**			

## INTRODUCTION

Cardioplegia is the most important component of myocardial protection during aortic cross-clamp in cardiac surgery with cardiopulmonary bypass (CPB). Currently, potassium-based cold blood cardioplegia is the most popular cardioplegia technique. In this technique, an induction dose is given for cardiac arrest, followed by multiple maintenance doses given at intervals for continued arrest. Blood cardioplegia (BC), as a type of extracellular cardioplegia, demonstrates its effect by depolarization of the myocardial membrane and inactivation of sodium channels. Extracellular cardioplegia solutions provide short arrest duration and, hereby, should be given in periods of 15-20 minutes for effective myocardial protection. Conversely, intracellular types of cardioplegia, which contain low sodium levels, show their effects by hyperpolarization and provide much longer arrest periods^[1,2]^.

Del Nido cardioplegia (DNC) is an extracellular type of crystalloid cardioplegia that consists of a 1:4 ratio of blood-crystalloid mixture solution. It is prepared with Plasma-Lyte, which has a similar amount of electrolyte with extracellular fluid. DNC cardioplegia exerts its effect by providing hyperkalemic arrest. Unlike BC, it contains magnesium, mannitol and lidocaine. While magnesium prevents accumulation of intracellular calcium ions, lidocaine blocks sodium channels and prolongs the duration of hyperpolarizing arrest. In addition, mannitol reduces myocardial edema by increasing osmolarity and removes free radicals. Although DNC cardioplegia is an extracellular cardioplegia, it can provide long periods of arrest. It provides cardioplegic arrest for more than 90 minutes with a single dose^[3,4]^.

Del Nido cardioplegia is well defined in pediatric heart surgery, and many new publications have been added to the literature regarding its usage in adult cardiac surgery^[5-7]^. Postoperative atrial fibrillation (PoAF) is a common complication after coronary artery bypass grafting (CABG). On-pump CABG has been shown to be associated with a higher incidence of PoAF than off-pump CABG procedure^[8]^. The aim of this study is to investigate the effect of DNC on the development of PoAF in patients undergoing isolated CABG.

## METHODS

### Patient Population

This retrospective observational clinical trial was conducted on patients who underwent on-pump isolated CABG at the Cardiovascular Surgery Department in Bursa Yuksek Ihtisas Training and Research Hospital between January and November 2019. Prior to June 2019, all adult patients received a BC solution. Our surgical team started using DNC in June 2019. Our study is a descriptive-correlational study and was approved by the local institutional Ethics Committee of the University of Health Sciences (approval number: 2011-KAEK-25 2019/10-19). All procedures were performed in accordance with the Declaration of Helsinki.

All data to be analyzed were collected retrospectively from our hospital medical records. Patients over 18 years old who underwent isolated on-pump CABG were included in the study. The patients were included in the study according to the exclusion criteria. First, we excluded chronic obstructive pulmonary disease (COPD) and valvular heart disease as risk factors that may lead to the development of atrial fibrillation (AF). Patients with preoperative AF or flutter, preoperative cerebrovascular disease, additional surgical intervention, peripheral arterial disease, renal failure, preoperative inotropic or mechanical support, redo surgery and emergency coronary surgery were not included in the study. All patients included in the study were using a beta-blocker drug due to coronary artery disease. Age, gender, history of hypertension, presence of diabetes mellitus, ejection fraction, body surface area, aortic cross-clamp time, total CPB time, number of anastomoses, and cardioplegia volume (hot-shot cardioplegia volume excluded) were recorded as data. In the postoperative period, need for inotropic medication (>5 µg/kg/min) more than 24 hours after surgery, need for intraaortic balloon pump (IABP), development of PoAF, length of intensive care unit (ICU) stay, death and hospital discharge time were added to the records. Death cases that occurred in the first 30 days after surgery were defined as operative mortality.

### Surgical and Myocardial Protection Procedure

Standard CPB with aorto-venous two-stage cannulation and mild hypothermia (32°C) was performed after median sternotomy. Heparin (300 IU/kg) was applied, and the activated clotting time was increased to over 400 seconds. In the blood cardioplegia group, cardiac arrest was achieved with cold blood cardioplegia (10-15 mL/kg, approximately 1000 ml), given antegrade, retrograde or both. Cardiac arrest was sustained with BC (300 ml), which was applied every 15-20 minutes. In addition, cardioplegia was delivered simultaneously via the anastomosed saphenous vein. In the DNC group, 1000 ml of cardioplegic solution was infused anterogradely as an initial dose. If the total aortic cross-clamp time was predicted to exceed 90 minutes, an additional dose (500 ml) was given 60 minutes after the initial dose. Both cardioplegia solutions were delivered at a temperature of 4°C. The content of the cardioplegia solutions is shown in Table 1.

**Table 1:** Characteristics of cardioplegia.

Components	Blood cardioplegia	Del Nido cardioplegia
Blood:Cardioplegia ratio	4:1	1:4
Base solution (mL)	Ringer Lactate (1000)	Isolyte-S (1000)
KCl (mEq)	16	26
8.4%, NaHCO_3_ (mL)	10	13
20%, mannitol (mL)	0	17
2%, lidocaine (mL/mg)	0	6.5/130
15%, MgSO_4_ (mL or g)	0	14/2

CPB was initiated at flow rates of 2-2.4 L/min/m^2^, with a roller pump with membrane oxygenator (Maquet, Getinge Group, Rastatt, Germany) and an arterial line filter. The distal anastomoses were performed during a single period of total aortic cross-clamp; however, the proximal ends were anastomosed under partial aortic clamping. Warm blood cardioplegia without potassium was infused in both groups just before the removal of the aortic cross-clamp. Protamine sulfate was used for heparin neutralization. Dopamine was the first choice when an inotropic agent was required while weaning from CPB. This inotropic support was maintained postoperatively for at least 24 hours. The patients were admitted to the cardiovascular surgery ICU after the operation. All patients received standard postoperative care. Following the provision of hemodynamic stability, extubation was performed at the earliest possible stage. Plasma troponin levels were measured in the morning of the first postoperative day (approximately 20 hours after surgery).

### Diagnosis of Postoperative Atrial Fibrillation

All patients were followed up in ICU by monitoring continuous heart rhythm and invasive blood pressure. In addition, a 12-lead electrocardiography (ECG) was also obtained daily during the ICU stay. Patients were monitored continuously with five-lead telemetry in the inpatient clinic. When patients complained of palpitations, dyspnea or angina, the 12-lead ECG was performed. AF was verified by a 12-lead ECG and diagnosed according to the guidelines of the European Society of Cardiology. An AF episode that lasted longer than 5 minutes was considered PoAF. Standard medical cardioversion therapy was performed with a 30-minute amiodarone infusion (5 mg/kg) followed by a maintenance dose of 900 mg/day.

### Statistical Analysis

In this study, Statistical Package for the Social Sciences (IBM SPSS Statistic Inc., version 21.0, Chicago, IL, USA) software was used for the statistical analysis of the data. Kolmogorov-Smirnov test and Shapiro-Wilk test of normality were used to identify the distribution of the variables. Continuous and ordinal variables are expressed as mean±standard deviation, and nominal variables are expressed as frequency and percentage. Student's t-test was used for continuous variables with normal distribution in both groups. Chi-square test was used in the comparison of the groups for nominal variables. In case the minimum expected count was <5, Fisher’s exact test was used. Mann-Whitney U test was used to compare the two groups for continuous variables without normal distribution. For all tests, *P*<0.05 was considered statistically significant. To evaluate whether there is a correlation between the development of PoAF and the length of hospital stay in the postoperative period, the point-biserial correlation coefficient test was performed, which correlates dichotomy data with continuously variable data. First, the relationship between the selected preoperative and operative independent variables and the development of PoAF was evaluated by univariate logistic regression analysis. Parameters with a significant *P*-value in univariate logistic regression analysis were included in the multivariate logistic regression analysis.

## RESULTS

In the present study, 255 patients were enrolled according to the exclusion criteria. A total number of 123 patients were recorded in the BC group (Group 1) (74% male, mean age 60.78±9.96 years) and 132 patients were recorded in the DNC group (Group 2) (80.3% male, mean age 61.67±10.16 years). The demographic characteristics and laboratory variables of the patients are summarized in Table 2. Both the BC group and the DNC group were similar in terms of demographic characteristics and laboratory variables.

**Table 2:** Demographic characteristics and laboratory variables of the patients.

	BC (Group 1)	DNC (Group 2)	*P*-value
	(n=123)	(n=132)	
Age (years)	60.78±9.96	61.67±10.16	0.483*
Male gender, n (%)	91 (74)	106 (80.3)	0.229^#^
Hypertension, n (%)	72 (58.5)	68 (51.5)	0.260^#^
Diabetes mellitus, n (%)	59 (48)	48 (36.4)	0.061^#^
Ejection fraction (%)	50.5±9.03	49.1±9.82	0.289^¶^
BSA	1.85±0.17	1.84±0.17	0.641*
Hematocrit (%)	40.0±5.77	40.3±4.48	0.646*
White blood cell (103/µL)	8.9±2.5	8.87±2.77	0.596^¶^
Platelet (103/µL)	243.80±66.94	245.20±64.70	0.545^¶^
Creatinine (mg/dL)	0.93±0.32	0.94±0.24	0.123^¶^
Na (mEq/L)	138.24±2.96	138.49±2.80	0.363^¶^
K (mEq/L)	4.25±0.51	4.27±0.43	0.728^¶^
Ca (mg/dL)	9.13±0.5	9.26±0.48	0.063^¶^
Mg (mg/dL)	1.89±0.18	1.92±0.21	0.434^¶^
Free T3 (pg/mL)	2.91±0.45	2.92±0.65	0.798^¶^
Free T4 (ng/dL)	1.25±0.21	1.35±0.46	0.235^¶^
TSH (IU/mL)	2.73±4.27	1.86±1.51	0.092^¶^
C-reactive protein (mg/dL)	8.54±11.93	9.77±19.90	0.967^¶^

* Student's t-test;

# Pearson chi-square test;

¶ Mann-Whitney U test. BC=blood cardioplegia; BSA=body surface area; DNC=del Nido cardioplegia

The operative and postoperative findings of the patients are summarized in Table 3. The number of anastomoses was greater in the DNC group, whereas aortic cross-clamp time and cardioplegia volume were greater in the BC group. These differences were statistically significant (*P*=0.005, *P*=0.042, *P*<0.001, respectively) (Table 3). In the postoperative period, the development of PoAF and hospital stay were greater in the BC group, and these findings were statistically significant (*P*=0.044, *P*<0.001, respectively) (Table 3). To evaluate whether there is a correlation between the development of PoAF and the length of hospital stay in the postoperative period, a point-biserial correlation coefficient test was performed, which correlates dichotomy data with continuously variable data. We detected a positive correlation between the development of PoAF and the length of hospital stay (r_pb_=0.149, *P*=0.018, point-biserial correlation coefficient). When the two groups were compared, there was no significant difference in terms of troponin T, inotropic drug support, need for IABP, and length of intensive care unit stay. The causes of mortality were recorded as stroke, low cardiac output syndrome and respiratory causes. There was no significant difference between groups in terms of causes of mortality (Table 3).

**Table 3:** Operative and postoperative parameters.

	BC (Group 1)	DNC (Group 2)	P-value
	(n=123)	(n=132)	
Number of anastomoses	3.32±0.91	3.67±0.92	0.005^¶^
Cross-clamp time (minutes)	62.8±18.66	59.30±17.36	0.042^¶^
Total CPB time (minutes)	90.01±24.25	95.16±23.48	0.086*
Cardioplegia volume (mL)	1697.56±264.71	1041.67±138.71	<0.001^¶^
Inotropic drugs support, n (%)	17 (13.8)	14 (10.6)	0.232^#^
IABP support, n (%)	14 (11.4)	10 (7.6)	0.298^#^
PoAF, n (%)	44 (35.8)	32 (24.2)	0.044^#^
ICU stay (days)	3.08±3.31	3.06±3.9	0.520^¶^
Hospital stay (days)	7.23±2.56	6.89±3.27	<0.001^¶^
Troponin T, ng/L	225±33.14	218.29±36.68	0.127*
Total mortality, n (%)	7 (5.7)	7 (5.3)	0.892^#^
LCOS, n (%)	2 (1.6)	2 (1,5)	1.000^a^
Stroke, n (%)	4 (3.3)	2 (1,5)	0.433^a^
Respiratory cause, n (%)	1 (0.8)	3 (2.3)	0.338^a^

* Student's t test;

# Pearson chi-square test;

¶ Mann-Whitney U test;

a Fisher's exact test. BC=blood cardioplegia; CPB=cardiopulmonary bypass; DNC=del Nido cardioplegia; IABP=intraaortic baloon pump; ICU=intensive care unit; LCOS=low cardiac output syndrome; PoAF=postoperative atrial fibrillation

In this study, risk factors related to the development of PoAF were included in the univariate logistic regression analysis. In the univariate logistic regression analysis, PoAF was significantly correlated with hypertension (OR 0.570; 95% CI 0.328-0.991; *P*=0.046) and cardioplegia volume (OR 1.001; 95% CI 1.000-1.001; *P*=0.048), but did not correlate with age, ejection fraction, C-reactive protein (CRP), total CPB time and aortic cross-clamp time (Table 4). Parameters that were correlated with PoAF in the univariate logistic regression analysis were evaluated in the multivariate logistic regression analysis. Although there was no correlation in the univariate analysis, the age parameter was included in the multivariate analysis since the role of advanced age in the development of AF has been shown in many studies^[9]^. Cardioplegia volume (OR 1.001; 95% CI 1.000-1.001; *P*=0.033) was identified as an independent predictor of PoAF development after CABG surgery in the multivariate analysis (Table 4).

**Table 4:** Binary logistic regression analysis to identify predictors of PoAF.

Variables	Univariate analysis			Multivariate analysis		
	*P*-value	Exp (B)	95% CI	*P*-value	Exp (B)	95% CI
		Odds ratio	Lower Upper		Odds ratio	Lower Upper
Age	0.062	1.026	0.999-1.055	0.207	1.019	0.990-1.048
HT	0.046	0.570	0.328-.991	0.301	0.734	0 .409-1.318
EF	0.417	0.988	0.961-1.017			
CRP	0.793	1.003	0.983-1.023			
Total CPB time	0.576	1.003	0.992-1.014			
Cross-clamp time	0.400	1.006	0.992-1.021			
Cardioplegia volume	0.048	1.001	1.000-1.001	0.033	1.001	1.000-1.001

CPB=cardiopulmonary bypass; CRP=C-reactive protein; EF=ejection fraction; HT=hypertension; PoAF=postoperative atrial fibrillation

## DISCUSSION

In previous studies, DNC has been shown to be as reliable as BC in adult cardiac surgery^[6,10-14]^. In this study, we evaluated the effects of DNC and BC on the development of PoAF in patients undergoing isolated CABG. While PoAF was observed, clinical results were also evaluated. We found that the aortic cross-clamp time (*P*=0.042) and the volume of delivered cardioplegia (*P*<0.001) were significantly lower in the DNC group and that the development of PoAF (*P*=0.044) and the length of hospital stay (*P*<0.001) were significantly higher in the BC group (Table 3). In multivariate logistic regression analysis, only higher cardioplegia volume was determined as an independent predictor for PoAF development (Table 4).

In previous studies comparing the results of standard cardioplegia and DNC, AF development was evaluated in the postoperative period, but no difference was found^[10-14]^. However, all of these studies included patients who had undergone either isolated heart valves or combined valve + coronary surgery, and risk factors for the development of AF were not excluded. In a previous study, Mariscalco et al.^[15]^ found PoAF rates for isolated CABG, valve surgery and combined surgery as 22.9%, 39.8% and 45.2%, respectively. Timek et al.^[6]^ and Çayır et al.^[12]^ did not report any difference in the development of PoAF in their studies, where they evaluated the effects of DNC in isolated coronary bypass patients. However, in both studies, accurate diagnosis of PoAF development was difficult, as patients with COPD were included in the study, and since a large-scale, retrospective case-control study has shown that patients with COPD had a 4-time higher risk for developing AF^[16]^. In their recent study, Timek et al.^[17]^ found that the development of PoAF was greater in the group of isolated coronary bypass patients using DNC. They concluded that this was due to the relatively higher Society of Thoracic Surgeons (STS) score in the del Nido group. For all these reasons, patients with COPD and those with valvular heart disease were not included in the present study to make our results more accurate and to not affect the outcome of the study. In addition, in our study, there was no difference between the groups in terms of known risk factors in AF development after CABG, such as advanced age, presence of hypertension (HT), and high CRP values.

In this study, we found that PoAF development and cardioplegia volume were significantly lower in the DNC group, and only higher cardioplegia volume was detected as an independent predictor for PoAF development in the multivariate logistic regression analysis. We can note that the previous studies mentioned in this discussion were related to the outcomes of the use of DNC and were not specific to PoAF development. The present study is adjusted for risk factors that affect AF development and is focused on evaluating the effect of DNC on PoAF development. Accordingly, it is a more specific study.

CPB is an important trigger of inflammatory response and a source of oxidative stress. While hypothermia, electrolyte imbalance, and agents used play a role in the onset of inflammatory response and tissue damage, myocardial ischemic cardioplegic arrest and reperfusion have been shown to be important triggers of tissue damage and inflammatory response^[18]^. The current inflammatory and systemic oxidative stress response caused by CPB can contribute to morbidity and mortality, causing endothelial damage in many systemic organs. Ischemic injury can lead to active neutrophil accumulation in the myocardium, which leads to the release of reactive oxygen species (ROS) and other proteolytic enzymes. Oxidative stress associated with CPB and cardioplegic arrest probably triggers cellular changes in the atrial tissue and cause impaired electrical activity. Atrial remodeling that develops ROS is responsible for the pathogenesis of PoAF^[19]^. On the other hand, ischemia during cardiac surgery is associated with decreased mitochondrial energy production, which can lead to changes in intracellular Na^+^, Ca^2+^ and pH^[19]^. Magnesium in the content of DNC inhibits the precipitation of calcium ions into the cell, which can reduce the oxidative stress response during ischemia. Consequently, it may have a negative effect on the development of PoAF by reducing the ROS formation.

In their study, Timek et al.^[17]^ found that aortic cross-clamp time was shorter in the del Nido group, although the average number of anastomoses was equal. Similarly, in our study, although the number of anastomoses was higher in the DNC group, aortic cross-clamp times were shorter (*P*=0.005). Although we did not plan to use DNC in patients who will receive more coronary anastomoses, we reached this finding as a result of the study. The reason for this is to apply a single dose of cardioplegia instead of administering cardioplegia every 15-20 minutes. The shorter ischemic time leads to less development of the oxidative stress response and related harmful reactions. This may suggest that DNC reduces PoAF development not only due to its electrolyte content, but also by shortening the ischemia time. We found that high cardioplegia volume is an effective factor on the development of PoAF in the logistic regression analysis (OR 1.001; 95% CI 1.000-1.001; *P*=0.033) (Table 4). Cardioplegia volume was statistically lower in the DNC group than in the BC group (*P*<0.001, Mann-Whitney U test). In the present study, this statistical result may elucidate the lesser development of PoAF in the DNC group. In previous studies, similar to our study, the amount of DNC used was significantly lower than BC^[11,14,17,20]^. This means less hyperpotassemic arrest. During hyperpotassemic cardioplegic arrest, membrane depolarization occurs, and a small quantity of Na and Ca channels proceed to work, and the amount of intracellular calcium increases. In contrast, the content of lidocaine in DNC increases Na^+^ channel blockade and prevents intracellular calcium accumulation, in addition to the effect of magnesium^[4]^. Additionally, the completion of coronary bypass with one-time cardioplegia provides good comfort to the surgeon.

Reliability is important in a new application or treatment option for myocardial protection in cardiac surgery. In meta-analyses^[21,22]^, it was stated that DNC has similar results to traditional cardioplegia and myocardial protection methods and is a good alternative to BC in routine adult cardiac surgery. When the studies by Timek et al.^[17]^, Yerebakan et al.^[20]^, Salinas et al.^[23]^ and Cayir et al.^[12]^, which were similar to our study, were evaluated, no difference was found between BC and DNC in terms of postoperative troponin levels and hospital mortality. Although our results were similar to these studies, our mortality rates were high. Stroke constituted 42.8% of the total mortality in the entire patient group. This condition increases mortality rates for both groups. In addition, these patients were over 75 years old and had HT and diabetes mellitus. Therefore, patients with stroke may have had clinically asymptomatic cerebrovascular disease, and circulatory failure may have developed during CPB. We found that the length of hospital stay was shorter in the DNC group, and the difference was statistically significant (*P*<0.001). We consider that this is due to PoAF, because significantly less PoAF was observed in the DNC group. We detected a positive correlation between the development of PoAF and the length of hospital stay (r_pb_=0.149, *P*=0.018, point-biserial correlation coefficient).

In our study, parallel to the meta-analyses, DNC was found reliable in isolated coronary bypass surgery. In contrast, Valooran et al.^[2]^ mentioned in their reviews that there are potential concerns about its use in adult cardiac surgery due to insufficient prospective randomized studies on DNC. In their technical articles on the use of DNC, Kim et al.^[24]^ mentioned that they did not use this solution in patients with coronary artery disease, because the adequate and uniform distribution of a single dose of cardioplegia to the myocardium cannot be guaranteed in the presence of a major vascular or microcirculatory disease. However, in all publications in the present article, it was stated that the use of DNC in coronary bypass surgery is safe.

### Limitations of the Study

There were several limitations of our study. First, the sample size was relatively small, and the study was retrospective and single-centered. Second, the measurement of cardiac output and the subsequent echocardiographic evaluation were not performed in the intensive care unit to evaluate postoperative myocardial functions. Therefore, myocardial evaluation adhered to the results of troponin levels, inotropic drug supplementation and need for IABP. Another limitation of the study was the exclusion of emergency and redo surgery cases. In comprehensive studies that include high-risk patients, it will be necessary to determine whether DNC provides adequate myocardial protection.

## CONCLUSION

In the present study, we investigated the effect of DNC and BC use on the development of PoAF in patients who underwent on-pump isolated CABG surgery. We found that the use of DNC leads to less PoAF development and provides shorter aortic cross-clamp time and shorter hospital stay. We found no difference in terms of myocardial protection and mortality. In the light of previous studies and our study, we think that single dose DNC can be safe and useful in CABG surgeries and that it has a lesser effect on PoAF development. However, further multicenter prospective randomized clinical trials are still required.

**Table:** 

Authors' roles & responsibilities	
USS	Substantial contributions to the conception or design of the work; or the acquisition, analysis, or interpretation of data for the work; drafting the work or revising it critically for important intellectual content; final approval of the version to be published
KKO	Substantial contributions to the conception or design of the work; or the acquisition, analysis, or interpretation of data for the work; drafting the work or revising it critically for important intellectual content; final approval of the version to be published
FT	Drafting the work or revising it critically for important intellectual content; final approval of the version to be published
SY	Drafting the work or revising it critically for important intellectual content; final approval of the version to be published

## References

[1] Chambers DJ (2003). Mechanisms and alternative methods of achieving cardiac arrest. Ann Thorac Surg.

[2] Valooran GJ, Nair SK, Chandrasekharan K, Simon R, Dominic C (2016). del Nido cardioplegia in adult cardiac surgery - scopes and concerns. Perfusion.

[3] Matte GS, del Nido PJ (2012). History and use of del Nido cardioplegia solution at Boston children’s hospital. J Extra Corpor Technol.

[4] O’Blenes SB, Friesen CH, Ali A, Howlett S (2011). Protecting the aged heart during cardiac surgery: the potential benefits of del Nido cardioplegia. J Thorac Cardiovasc Surg.

[5] Mick SL, Robich MP, Houghtaling PL, Gillinov AM, Soltesz EG, Johnston DR (2015). del Nido versus Buckberg cardioplegia in adult isolated valve surgery. J Thorac Cardiovasc Surg.

[6] Timek T, Willekes C, Hulme O, Himelhoch B, Nadeau D, Borgman A (2016). Propensity matched analysis of del Nido cardioplegia in adult coronary artery bypass grafting: initial experience with 100 consecutive patients. Ann Thorac Surg.

[7] Ziazadeh D, Mater R, Himelhoch B, Borgman A, Parker JL, Willekes CL (2017). Single-dose del Nido cardioplegia in minimally invasive aortic valve surgery. Semin Thorac Cardiovasc Surg.

[8] Bohatch Júnior MS, Matkovski PD, Di Giovanni FJ, Fenili R, Varella EL, Dietrich A (2015). Incidence of postoperative atrial fibrillation in patients undergoing on-pump and off-pump coronary artery bypass grafting. Rev Bras Cir Cardiovasc.

[9] De Jong MJ, Morton PG (2000). Predictors of atrial dysrhythmias for patients undergoing coronary artery bypass grafting. Am J Crit Care.

[10] Ad N, Holmes SD, Massimiano PS, Rongione AJ, Fornaresio LM, Fitzgerald D (2018). The use of del Nido cardioplegia in adult cardiac surgery: a prospective randomized trial. J Thorac Cardiovasc Surg.

[11] Koeckert MS, Smith 3rd DE, Vining PF, Ranganath NK, Beaulieu T, Loulmet DF (2018). Del Nido cardioplegia for minimally invasive aortic valve replacement. J Card Surg.

[12] Cayir MC, Yuksel A (2020). The use of del Nido cardioplegia for myocardial protection in isolated coronary artery bypass surgery. Heart Lung Circ.

[13] Hamad R, Nguyen A, Laliberté É, Bouchard D, Lamarche Y, El-Hamamsy I (2017). Comparison of del Nido cardioplegia with blood cardioplegia in adult combined surgery. Innovations (Phila).

[14] Kim JS, Jeong JH, Moon SJ, Ahn H, Hwang HY (2016). Sufficient myocardial protection of del Nido cardioplegia regardless of ventricular mass and myocardial ischemic time in adult cardiac surgical patients. J Thorac Dis.

[15] Mariscalco G, Engström KG (2009). Postoperative atrial fibrillation is associated with late mortality after coronary surgery, but not after valvular surgery. Ann Thorac Surg.

[16] Banach M, Rysz J, Drozdz JA, Okonski P, Misztal M, Barylski M (2006). Risk factors of atrial fibrillation following coronary artery bypass grafting: a preliminary report. Circ J.

[17] Timek TA, Beute T, Robinson JA, Zalizadeh D, Mater R, Parker JL (2019). Del Nido cardioplegia in isolated adult coronary artery bypass surgery. J Thorac Cardiovasc Surg.

[18] Zakkar M, Ascione R, James AF, Angelini GD, Suleiman MS (2015). Inflammation, oxidative stress and postoperative atrial fibrillation in cardiac surgery. Pharmacol Ther.

[19] Suleiman MS, Zacharowski K, Angelini GD (2008). Inflammatory response and cardioprotection during open-heart surgery: the importance of anaesthetics. Br J Pharmacol.

[20] Yerebakan H, Sorabella RA, Najjar M, Castillero E, Mongero L, Beck J (2014). Del Nido cardioplegia can be safely administered in high-risk coronary artery bypass grafting surgery after acute myocardial infarction: a propensity matched comparison. J Cardiothorac Surg.

[21] An KR, Rahman IA, Tam DY, Ad N, Verma S, Fremes SE (2019). A systematic review and meta-analysis of del Nido versus conventional cardioplegia in adult cardiac surgery. Innovations (Phila).

[22] Li Y, Lin H, Zhao Y, Li Z, Liu D, Wu X (2018). Del Nido cardioplegia for myocardial protection in adult cardiac surgery: a systematic review and meta-analysis. ASAIO J.

[23] Guajardo Salinas GE, Nutt R, Rodriguez-Araujo G (2017). Del Nido cardioplegia in low risk adults undergoing first time coronary artery bypass surgery. Perfusion.

[24] Kim K, Ball C, Grady P, Mick S (2014). Use of del Nido cardioplegia for adult cardiac surgery at the Cleveland clinic: perfusion implications. J Extra Corpor Technol.

